# AgNPs biosynthesized from *Pseudomonas* Z9.3 metabolites as antimicrobial agents against bacterial and fungal pathogens

**DOI:** 10.3389/fmicb.2025.1565689

**Published:** 2025-04-07

**Authors:** Svitlana Plokhovska, Elena Fuente-González, Enrique Gutierrez-Albanchez, Francisco Javier Gutierrez-Mañero, Beatriz Ramos-Solano

**Affiliations:** ^1^Faculty of Pharmacy, Universidad San Pablo-CEU Universities, Madrid, Spain; ^2^Department of Cell Biology and Biotechnology, Institute of Food Biotechnology and Genomics NAS of Ukraine, Kyiv, Ukraine

**Keywords:** biological synthesis, beneficial bacteria, silver nanoparticles, pathogens, antimicrobial activity

## Abstract

**Introduction:**

An eco-friendly method for the biosynthesis of functional silver nanoparticles (AgNPs) using plant growth-promoting bacteria (PGPB), specifically *Pseudomonas* sp. Z9.3, has been developed. The growing need for sustainable and non-toxic nanoparticle production makes this method significant for various applications.

**Methods:**

The influence of physicochemical parameters, such as temperature, pH, and concentrations of AgNO_3_, on the synthesis of AgNPs was studied. The formation of AgNPs was confirmed by UVvis, SEM/TEM, FTIR, and XRD analysis. Antibacterial activity was assessed using the antibacterial disk diffusion assay. For antifungal activity, AgNPs were added to the agar medium, and the size of the inhibition zone was measured.

**Results and discussion:**

Two optimal conditions were identified: 37°C, pH 9, and a 5:1 ratio of bacterial supernatant to 5 mM AgNO_3_ (S1-9), and 37°C, pH 7, with a 2:4 ratio (S4-7). The UV-visible spectroscopy results showed an absorption range between 400 and 450 nm, confirming the formation of AgNPs. The SEM and TEM analysis showed the spherical shape of AgNPs with a good distribution of nanoparticles and the average size ranged from 8.24 ± 0.26 to 13.32 ± 0.4 nm. Antibacterial activity against different pathogenic bacteria and fungi was tested. Antibacterial activity of AgNPs against six human pathogens and three phytopathogens was evaluated. The antibacterial potential of S1-9 against Gram-negative strains was lower than against Gram-positive strains; in particular, *S. epidermidis* was the most sensitive (93.76%) compared to the equivalent concentration of Ag. In the case of fungi, S4-7 exhibited better inhibitory activity compared to the negative control. The highest dose (120 ppm) of S4-7 AgNP inhibited fungal growth being the most sensitive *Alternaria* sp. (74.97%), followed by *Stemphylium* sp. (66.30%), *Fusarium* sp. (45.62%), and *Rhizopus* sp. (32.68%). These findings highlight the potential of synthesized AgNPs as antimicrobial agents for both bacterial and fungal pathogens.

## 1 Introduction

Nanotechnology has attained remarkable attentiveness in recent years due to extensive applications in diverse fields. The advancements in nanotechnology have enabled the production of nanoparticles (NPs) which have many different applications such as textiles, electronics, machinery, medicine, food industry and agriculture (Dikshit et al., 2021; Mgadi et al., 2024). These NPs have numerous unique properties attributed to their small size (< 100 nm). The size of AgNPs is key to their effectiveness in agriculture. Nanoparticles smaller than 60 nm are ideal for penetrating plant tissues, as they can pass through the stomata (10–60 μm) and plasmodesmata (20–50 nm). Spherical particles are especially effective for penetration and distribution in plant tissues (Yadav et al., 2024). This makes them good candidates for use in agriculture as biosensors, fertilizers, herbicides, bactericides and fungicides (Hazarika et al., 2022; Mgadi et al., 2024). Nowadays, silver nanoparticles (AgNPs) are one of the most popular materials due to the unique physicochemical and biochemical features. Owing to their small size and large surface area, AgNPs have high antibacterial activity (Huq et al., 2022), against both Gram-positive and Gram-negative strains (More et al., 2023; Nie et al., 2023). Additionally, the antifungal activity of AgNPs has been shown to depend on their surface properties, including the functional groups present (Matras et al., 2022; Ribeiro et al., 2023). These findings highlight the importance of the physicochemical parameters of AgNPs in their fungicidal potential.

AgNPs exhibit antimicrobial effects through several mechanisms. Their small size and high surface area allow them to interact with microbial cell membranes, causing structural damage and leakage of cellular contents. Additionally, AgNPs can generate reactive oxygen species (ROS) within microbial cells, inducing oxidative stress that further damages cellular components such as lipids, proteins, and DNA. Moreover, AgNPs can bind to the bacterial cell wall, interfering with cell wall synthesis and affecting cellular respiration (Rai et al., 2021; Mussin and Giusiano, 2022). While AgNPs show strong antimicrobial potential, their toxicity raises concerns, especially in plants. At high concentrations, AgNPs can lead to oxidative stress, membrane damage, and disruption of metabolic pathways, which can be toxic to plants (Yang et al., 2019). However, at low concentrations, AgNPs can enhance seed germination, promote early growth, stimulate defense mechanisms, and increase nutrient absorption (Noori et al., 2024; Guzmán-Báez et al., 2021). Further research is needed to determine safe concentrations and assess environmental impacts.

In general, NPs are synthesized using physical and chemical methods, orientated to reduction of the chemical element. However, these methods are hazardous and cause significant environmental problems (Saravanan et al., 2021). AgNPs can also be synthesized by biological means using different plant, bacteria, fungi, yeast and algae. This process relies on natural antioxidant compounds present in natural materials, or enzymes released by organisms. The biological method for NP synthesis is a better alternative as it is eco-friendly, cost-efficient and requires less time and energy (Koul et al., 2018; Raj et al., 2022). Different biological agents react differently with metal solutions during NPs formation. In recent years, many reports on the biosynthesis of AgNPs from various bacterial species have been shown (Ansari and Rehman, 2021; Nie et al., 2023; Siddiqi et al., 2018). Microbes produce different organic materials and, therefore, the underlying mechanism to reduce metal ions differs from one organism to another. Metal ions are reduced to NPs because of the presence of enzymes synthesized by the microbes (Ovais et al., 2018), or else, because of bacterial metabolites (Srimathi et al., 2024). A significant advantage of the biological synthesis is that the reduction process coats the metal with organic matter, which stabilizes the NP and contributes to it improve its effect (Hulkoti and Taranath, 2014). Therefore, using metabolites from specific bacterial strains will lead to the production of unique AgNPs, as each strain releases distinct metabolites that influence the synthesis process and the properties of the NPs. Biological synthesis generates AgNPs with a high surface-to-volume ratio, which gives them remarkable chemical and biological properties. Both the atoms constituting the NPs and the molecules located on their surface are highly reactive and interact with other molecules, such as those in plant tissues, soil particles, and microbial communities. This characteristic allows the NPs to effectively act as carriers of nutrients and bioactive compounds, penetrating plant cell walls, stomata and plasmodesmata (Yadav et al., 2024).

An interesting group of bacteria for this purpose are the Plant Growth Promoting Bacteria (PGPB). These bacteria contribute to plant fitness through different mechanisms like nitrogen fixation, phosphate solubilization, phytohormones production, soil organic matter mineralization, and phytopathogens suppression, etc. Microbes such as *Pseudomonas, Bacillus, Azotobacter, Enterobacter, Azospirillum, Alcaligenes* and *Klebsiella* have been reported to enhance plant growth (Mohanty et al., 2021), protect plants by activation of plant immune system (Martin-Rivilla et al., 2020) or directly fighting pathogens in the plant surroundings by biocontrol (Jiao et al., 2021); moreover, the ability of bacterial metabolites to mimic bacterial effects has also been demonstrated (Martin-Rivilla et al., 2019, 2021). Therefore, based on the above, the synthesis of NPs using PGPB metabolites is an exciting approach, as the potential of these metabolites can be boosted based when coats the metal ion generating the NPs. The development of a conjugative approach of NPs with PGPB metabolites offers enormous potential to improve both plant yield and disease resistance (Fadiji et al., 2022; Kalaimurugan et al., 2019; Mandal and Sarkar, 2024; Plokhovska et al., 2025). Despite the potential benefits of metal-based NPs synthesized from beneficial bacteria metabolites for agricultural applications, there is a significant barrier due to the lack of understanding regarding their precise mechanisms of action, selectivity, and toxicity (Fadiji et al., 2022).

Based on the above, we hypothesized that metabolites from the beneficial strain *Pseudomonas* Z9.3 would be able to reduce silver ions, resulting in AgNPs with unique properties due to the distinctive metabolites produced by Z9.3. As bacterial and fungal pathogens threaten not only human health but also compromise agronomic yield, this uniqueness makes them an excellent material for agriculture (Mandal and Sarkar, 2024). Hence, the present work explores the biological synthesis of AgNPs using beneficial strain of *Pseudomonas* Z9.3 for potential use to inhibit growth of bacterial and fungal pathogens as causative agents of human and plant diseases. To achieve this objective, the best conditions for biological synthesis, physicochemical characterization and *in vitro* antibacterial and antifungal activity was evaluated.

## 2 Materials and methods

### 2.1 Preparation of *Pseudomonas* sp. strain culture

*Pseudomonas* Z9.3 was isolated from the rhizosphere of *Pinus pinea* in Segovia, Spain (Garcia-Villaraco et al., 2024). The bacterial strain was periodically cultured and maintained in plate count agar (PCA). For biosynthesis of AgNPs, *Pseudomonas* sp. was cultured in 500 mL of nutrient broth (NB) medium. The bacterial strain was incubated at 28°C in an orbital shaker at 150 rpm for 24 h. The bacterial culture was centrifuged at 5,000 rpm for 20 min using a refrigerated centrifuge (AFI LISA MultiLab Centrifuge, France). The pellet was discarded, and the supernatant was filtered by 0.25 mm (VWR International, USA) and used for AgNPs biosynthesis. Cell-free culture supernatant of *Pseudomonas* Z9.3 was confirmed, as no bacterial growth after incubating 100 μL of supernatants on nutrient agar at 28°C for 24 h was detected.

### 2.2 Biological synthesis of AgNPs

The culture supernatant and 5 mM AgNO_3_ aqueous solution were mixed in a different ratio (supernatant/AgNO_3_, v/v; 5:1, 4:2, 3:3, 2:4, 1:5) to determine the optimal concentration to produce AgNPs; supernatants were brought to pH 7 and 9 before mixing with AgNO_3_. The flasks were incubated for 24 h in an orbital shaker at 150 rpm; nucleation temperature was set at 28°C or 37°C. Nutrient broth without AgNO_3_ solution was used as control. After incubation period, the AgNPs were collected by centrifugation at 5,000 rpm for 20 min (AFI LISA MultiLab Centrifuge, France) and washed thoroughly with miliQ water to remove the unconverted metal ions or any other constituents, stopping nucleation. The presence of NPs was confirmed using UV, TEM/SEM, FTIR, and XRD analysis as described below. The purified AgNP were freeze-dried and lyophilized (Cryodos, Telstar, Spain) to obtain a powder, which was dissolved in 1 mL distilled water to prepare a stock solution (4,000 ppm) for further characterization and application in antimicrobial activity experiments.

### 2.3 Characterization of synthesized AgNPs

The bioreduction of the Ag^+^ ions in the solution was observed by changes in color from light yellow to dark brown. The absorption spectrum of this solution was monitored by UV–Visible spectrometry from 200 to 800 nm at 1 nm resolution using a SPECTROstar Nano spectrometer (BMG LABTECH, Germany; RRID:SCR_019751) confirming presence of Ag^0^ by increased absorption at 420–450 nm.

Size distribution and morphology of synthesized AgNP were estimated by Transmission Electron Microscope (Jeol JEOL JEM-1400Flash; RRID:SCR_020179). The measurements have been carried out by depositing a few drops of the sample in suspension on aluminum and magnesium sample holders, allowing it to dry before placing it in the microscopes. In the black and white images, topography, we used the thermoelectrically cooled SDD (Silicon Drift Detector) with an acceleration voltage ranging from 40 kV to 120 kV, in steps of 33 V. Analyses were carried out at ICTS-CNME (https://cnme.es/).

Functional groups responsible for the synthesis and stabilization of AgNPs were detected by Fourier transform infrared (FTIR) spectroscopy, on KBr pellets. The samples were scanned using Bruker IFS66V FTIR Spectrometers (Bruker, USA) with a resolution of 4 cm^−1^ and a range of 450–4,000 cm^−1^. Analyses were carried out at SIDI (https://www.uam.es/uam/en/sidi/unidades-de-analisis/unidad-analisis-estructural-molecular/ftir).

The lyophilized samples of the biosynthesized AgNPs coated on X-ray diffraction (XRD) grids were studied for XRD patterns using a Bruker D8 diffractometer (RRID:SCR_019761) and a fast detector LynxEye, operating at a voltage of 40 kV and current of 40 mA with a scan rate of 0.01/s (https://www.uspceu.com/investigacion/servicios-apoyo/servicio/difracci%C3%B3n-de-rayos-x).

### 2.4 Antibacterial activity of AgNPs

The antibacterial disk diffusion assay on antibacterial activity of biosynthesized AgNPs was evaluated using the Kirby-Bauer technique (Bauer et al., 1966). Three Gram-negative (*Salmonella* sp., *E. coli* and *P. aeruginosa*) and three Gram-positive (*Enterococcus* sp., *S. epidermidis* and *S. aureus*) human pathogen bacterial strains were used in this study. Also, three phytopathogenic bacteria were tested in this study: *X. campestris* pv *oryzae, X. campestris* pv *tomato* and *P. syringae* DC3000.

Mueller Hinton Agar (MHA) was used for the analysis of antibacterial activity on disposable sterile Petri dishes and stored in the refrigerator at 4°C for further use. Gentamicin (10 μg/mL), AgNO_3_ 5 mM solution in equivalent concentrations to NP, and cell-free culture supernatant of *Pseudomonas* Z9.3 were used as positive and negative controls. The bacterial pathogen suspension was prepared by subculturing bacteria into NB medium. The human pathogenic bacteria were incubated for 24 h in orbital shaker at 150 rpm at 37°C; phytopathogenic bacteria were incubated at 28°C. The optical density OD600 of the bacterial suspension was adjusted to 0.1 absorbance at 600 nm, that corresponds to 1.5 × 106 cfu/mL. One hundred microliter of the suspension were then spread evenly over the MHA plate using a sterile disposable spatula. Paper disks (6 mm) containing 30 μL of the AgNPs at different concentrations (10, 25, 50, 75, and 100%) were placed on the center of the petri dishes. The diameter of the growth inhibition halo of each bacteria was measured by digital image analysis using ImageJ software (Version 1.8.0_345; RRID:SCR_003070). The experiment was carried out in triplicate and measurements were made after 24 h of incubation at 37°C for human pathogens while phytopathogens were incubated at 28°C. The results were recorded as the mean ± standard deviation of the triplicate experiment, relative to the positive control. Growth inhibition was calculated as:


$$\begin{array}{l} \text{Inhibition }\left(\%\right)=\frac{\text{DCD }-\text{ DTD}}{\text{DCD}}\;\times 100\;\% \end{array}$$

where, DCD—diameter of the control disk with AgNO_3_, mm; DTD—diameter of the NPs treatment disk, mm. Each treatment had three replicates.

### 2.5 Antifungal activity of AgNPs

The effect of AgNPs was studied on *Fusarium* sp., *Stemphylium* sp., *Alternaria* sp. and *Rhizopus* sp. fungi. The Potato-Dextrose-Agar (PDA) culture medium was prepared, sterilized and stored in the refrigerator at 4°C for further use. AgNP were added to the medium at concentrations of 30, 60, and 120 ppm. A PDA disk with fresh fungal mycelium of 0.5 cm in diameter, was placed in the center of each Petri dish with NPs in the medium; plates were incubated at room temperature, in the dark, until mycelial growth in controls covered the surface, variable according to each fungus. The diameter of the growth of each fungus was measured by digital image analysis using ImageJ software (Version 1.8.0_345; RRID:SCR_003070). The mycelial growth was measured in every Petri dish after 5 days after incubation for *Alternaria* and *Stemphylium* spp. and 12 days after incubation for *Fusarium* and *Rhizopus* spp. The percentage of mycelial growth inhibition was calculated using the following equation proposed by Vera-Reyes et al. (2022):


$$\begin{array}{l} \text{Inhibition }\left(\%\right)\;=\;\frac{\text{DCC }-\text{ DPC}}{\text{DCC}}\;\times 100\;\% \end{array}$$

where, DCC—diameter of the control colony, mm; DPC—diameter of the problem colony (fungus in the presence of AgNP, mm). Each treatment had three replicates.

### 2.6 Statistical analysis

All antimicrobial experiments were conducted in three independent replicated and the results were stated as the mean ± standard deviation (mean ± SD). Data was analyzed by one-way analysis of variance (ANOVA). The statistical significance of differences between groups was assessed using *p*-values, with values of *p* < 0.05 considered statistically significant. When differences were significant according to ANOVA, an LSD test was applied to identify which groups showed significant differences, by evaluating the pairwise differences in group means. Values of *p* < 0.05 indicated the significant differences. Different letters indicate significant differences between treatments. The statistical data analysis was analyzed using IBM SPSS Statistics (Version 29.0.2.0; RRID:SCR_016479).

## 3 Results

### 3.1 Characterization of synthesized AgNPs

**Figure 1:**
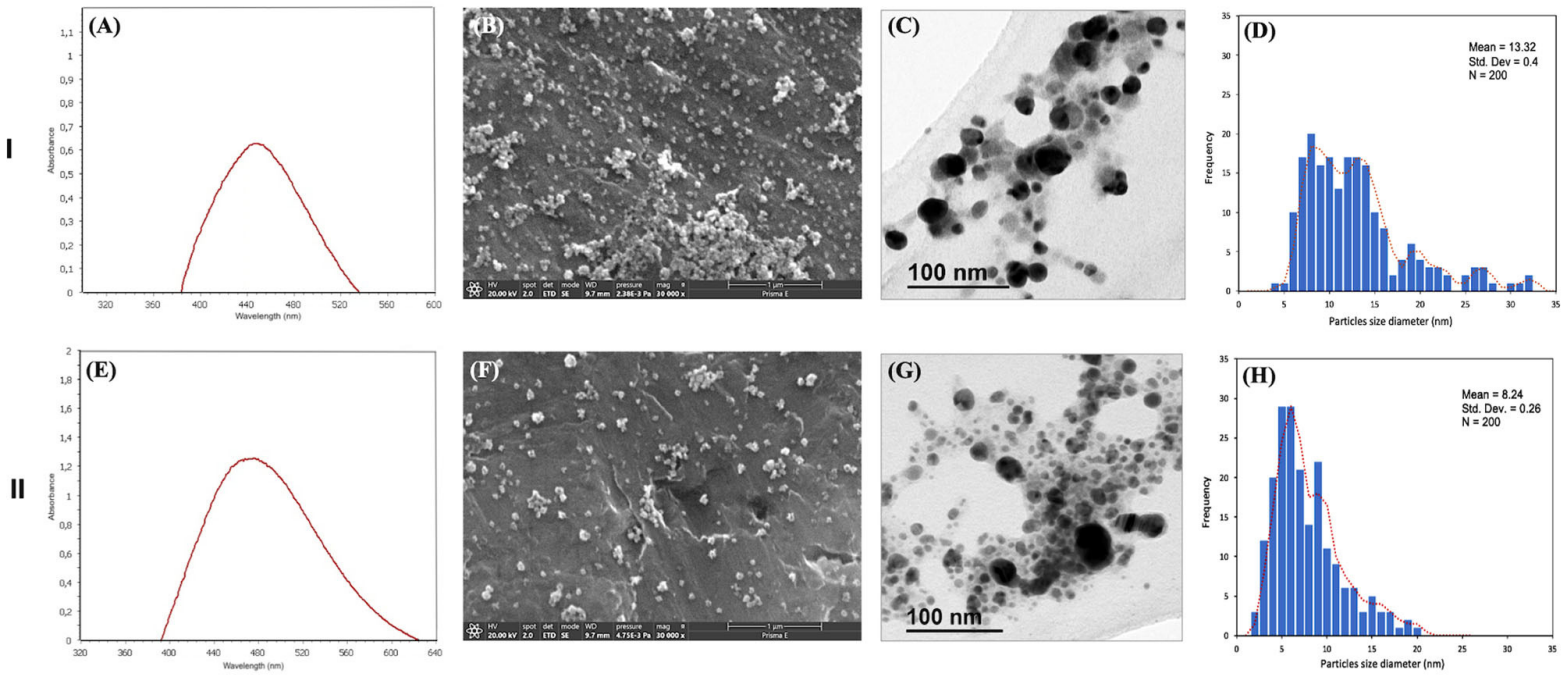
Characteristics of the biosynthesized AgNPs (I–S1-9; II–S4-7): **(A, E)** UV-vis spectra; **(B, F)** SEM images; **(C, G)** TEM images; **(D, H)** histogram of the particle size distribution of NPs. Bar: 1 μm for SEM and 100 nm for TEM.

AgNPs were successfully bio-synthesized after mixing bacterial metabolites contained in the supernatant with 5 mM AgNO_3_ in different volumes (working solution). The AgNPs synthesis was indicated by the change of color from light yellow to dark brown of the working solution. The brown color intensity of working solution increased with higher volumes of AgNO_3_ as shown by the different volume ratios results [S1 5:1, S2 4:2, S3 3:3, S4 2:4, S5 1:5 (supernatant:AgNO_3_ 5mM;)]. The UV-vis spectra revealed peaks between 400 and 450 nm, corresponding to reduced silver. The UV-vis spectra of AgNPs synthesized at different pH (7–9), nucleation temperatures (28°C and 37°C), and volume ratios [S1 5:1, S2 4:2, S3 3:3, S4 2:4, S5 1:5 (supernatant:AgNO_3_ 5 mM)] were studied. Two conditions, S1 volume ratio with pH9 (S1-9) and S4 volume ratio with pH7 (S4-7), both bio-synthesized at 37°C, were selected for further study, considering the different intensities and peaks recorded at the different ratios (Figures 1A, E). We conducted a SEM/TEM analysis of the two selected samples: S1-9 (Figures 1B, C, respectively) and S4-7 (Figures 1F, G, respectively). The results indicated that the shape of the bio-biosynthesized AgNPs was mostly spherical, and that the particle size was ranging from 4 to 32 nm with an average size of 13.32 ± 0.4 nm for S1-9 (Figure 1D), and from 2 to 20 nm, with an average size of 8.24 ± 0.26 nm for S4-7 (Figure 1H).

**Figure 2:**
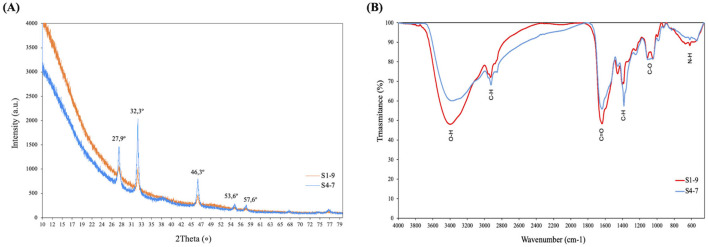
Characteristics of biosynthesized AgNPs (S1-9/S4-7): **(A)** XRD analysis; **(B)** FTIR spectra.

The crystalline nature of the bio-synthesized AgNPs was determined using XRD analysis (Figure 2A). Diffraction peaks were observed in the bio-synthesized AgNPs at 27,9°, 32,3°, 46,3°, 53,6° and 57,6°, a profile associated to a face-centered cubic structure of AgCl crystals (Ref. no. 31-1238). S1-9 and S4-7 had the same peaks which differed only in the intensity level of these peaks, which were more intense for S4-7 AgNP.

The components that coated the silver ion during the AgNPs (S1-9 and S4-7) biosynthesis (AgNPs surface molecules) were analyzed. The presence of functional groups of nutrient broth and bacterial metabolites (supernatant) of *Pseudomonas* Z9.3 was identified by FTIR spectroscopy (Supplementary Figure S1). FTIR spectra of AgNPs (S1-9 and S4-7) showed all the characteristic peaks present in supernatant (Figure 2B). The major band at 3,375 cm^−1^ is attributed to O–H stretching vibrations; 2,936 cm^−1^ is attributed to C–H stretching of an aromatic compound; the distinctive peak at 1,652 cm^−1^ is attributed to C=O carbonyl group and C=C stretching vibrations; the bands at 1,452, 1,335, 1,247, 1,120, and 1,082 cm^−1^ are attributed to C–H bonds and C–O stretching vibrations, O–H deformation vibrations, and C–O stretching vibrations due to the protein, respectively; the band at 620 cm^−1^ is attributed to N–H stretching of the nitro compound (Table 1). The presence of these groups confirms the presence organic compounds (metabolites of *Pseudomonas* Z9.3) and suggests that these functional groups reduce Ag^+^ and contribute to AgNPs stabilization forming an organic matter coating.

**Table 1:** FTIR analysis of the functional group of AgNPs for the two studied samples.

**Wavenumber (cm** ^**−1**^ **)**			**Vibrational assignment**
Filtrate Z9.3	S1-9 NPs	S4-7 NPs	
3374.87	3398.85	3372.83	O–H stretching vibrations
2935.56	2929.23	2925.75	C–H stretching of an aromatic compound
1652.37	1638.02	1638.62	C=O carbonyl groups and C=C stretching
1452.83	1457.18	1453.66	C–H bonds and C–O stretching vibrations
1401.72	1403.42	–	
1335.10	1387.11	1384.36	O–H deformation vibrations
1246.82	1247.10	1247.22	C–O stretching vibrations due to the protein
1119.57	1113.44	1107.79	
1082.23	1047.75	1051.91	
993.47	995.21	986.18	C=C–H bonds
921.28	923.40	924.50	
872.88	–	832.44	C–H stretching vibrations
619.80	620.07	620.11	N–H stretching of the nitro compound

### 3.2 Antimicrobial activity of AgNPs

Antimicrobial activity was tested on human pathogenic bacteria, phytopathogenic bacteria and phytopathogenic fungi. The antibacterial activity of AgNPs was investigated against the following human pathogenic bacteria, *S. aureus, S. epidermidis, Enterococcus* sp*., Salmonella* sp*., P. aeruginosa* and *E. coli*. The potential antibacterial activity of biosynthesized AgNPs against human pathogenic bacteria was confirmed by the formation of an inhibition zone, for which the diameter was measured (Supplementary Table S1); the inhibition capacity of an equivalent AgNO_3_ concentration was also evaluated to confirm that the observed effects were not only due to silver ions. Results are expressed as growth inhibition zone of AgNPs compared to growth inhibition zone by an equivalent AgNO_3_ concentration.

**Figure 3:**
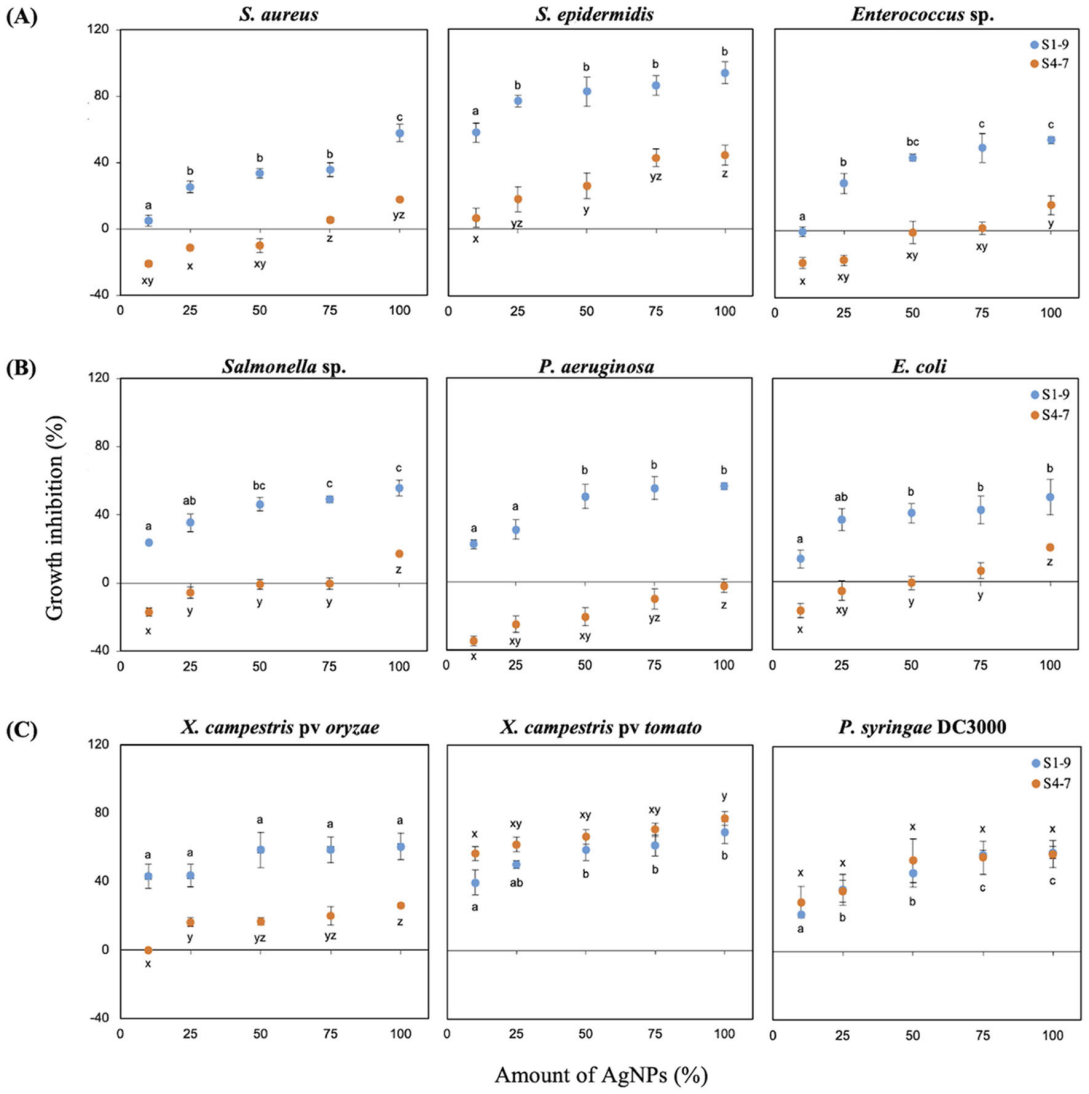
Antibacterial activity of AgNPs relative to the positive control Ag: **(A)** growth inhibition (%) of Gram-positive bacteria and **(B)** Gram-negative bacteria; **(C)** phytopathogenic bacteria. The results were recorded as mean ± SD of a triplicate experiment. Different letters (a, b, c for S1 and x, y, z for S4) indicate significant differences between treatments, as determined by the ANOVA test (*p* < 0.05).

Synthesized AgNPs showed antibacterial activity against both types of Gram-positive and Gram-negative human pathogenic bacteria, and against phytopathogenic bacteria (Figure 3). Antibacterial effect was strain-dependent and dose-dependent; S1-9 AgNPs showed higher activity than S4-7 AgNPs for all of them. The Gram-positive *S. epidermidis* was the most sensitive strain to both AgNPs; in particular, growth inhibition ranged from 7 to 44% for S4-7 AgNPs, and from 58 to 94% for S1-9 AgNPs; among Gram-positive bacteria and based on the lowest concentration of S1-9 AgNP tested, the most sensitive was *S. epidermidis* > *S. aureus* > *Enterococcus* species (Figure 3A), and, among the Gram-negative, *Salmonella sp*. > *E. coli* > *P. aeruginosa* (Figure 3B).

Antibacterial activity against various phytopathogenic bacteria (*X. campestris* pv *oryzae, X. campestris* pv *tomato, P. syringae* DC3000) was tested and expressed as described for human pathogenic bacteria (Figure 3C). Growth inhibition by NPs was dependent on the bacterial species and more intense than the equivalent AgNO_3_ concentration (Supplementary Table S2). *X. campestris* pv *oryzae* was sensitive to both NPs being S1-9 more effective (43–61% inhibition), while a low effect was shown for S4-7 (16–26% inhibition). Both types of AgNPs inhibited growth of *X. campestris* pv *tomato* similarly, from 40 to 60% for S1-9, and from 57 to 77% for S4-7. A similar result was found for *P. syringae* DC3000, with growth inhibition ranging from 22 to 59%, depending on the concentration of either AgNP, S1 or S4.

**Figure 4:**
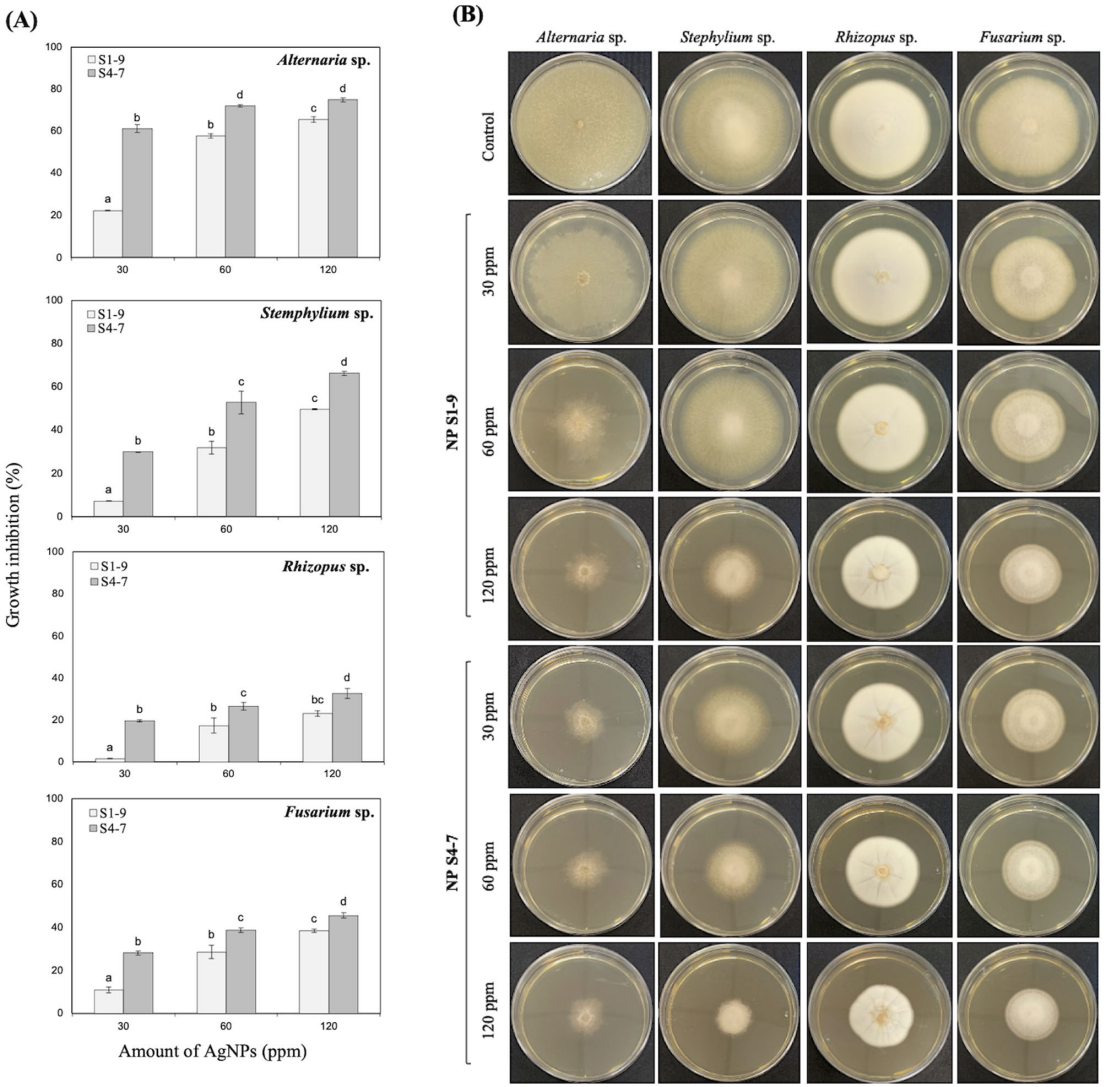
**(A)** Percentage inhibition of radial growth of phytopathogenic fungi strains; results were recorded as mean ± SD of a triplicate experiment. Statistical significance was determined by one-way ANOVA (*p* < 0.05); different letters indicate differences between treatments. **(B)** Petri dish demonstrating the growth inhibition zone of fungi at different AgNPs concentration.

Antifungal activity against various phytopathogenic fungi (*Alternaria* sp., *Stemphylium* sp., *Rhizopus* sp. and *Fusarium* sp.) was tested. The results showed that the inhibition of the mycelial growth caused by AgNPs varies depending on the fungal species, concentration and type of AgNPs (Figures 4A, B). Both types of AgNPs significantly inhibited growth for all tested fungal strains but S4-7 was more effective. Fungal growth inhibition ranged from 20 to 61% in *Alternaria* and *Stemphylium*, and from 5 to 40% for *Fusarium* and *Rhizopus*; inhibition was detected even at the lowest concentration tested (30 ppm S4-7). With the highest dose (120 ppm), the most sensitive was *Alternaria* sp. (74.97 ± 0.94% growth inhibition), followed by *Stemphylium* sp. (66.3 ± 0.95%), *Fusarium* sp. (45.62 ± 1.21%) and *Rhizopus* sp. (32.68 ± 2.37%). Figure 4B shows the mycelial growth of pathogenic fungi at different AgNPs concentrations.

In summary, S1-pH9 and S4-pH7 AgNPs inhibited the growth of all human pathogenic bacteria, phytopathogenic bacteria and phytopathogenic fungi, and were more effective compared to the equivalent AgNO_3_ concentration.

## 4 Discussion

Biological synthesis of nanomaterials offers an excellent alternative for producing non-toxic nanomaterials with minimal waste. Biosynthesis of metal-based NPs can be achieved with several biological substrates capable of reducing metal salts. When plant extracts are used, the process is termed green synthesis. In this approach, the development of NPs depends on the phytochemicals such as ascorbic acid, alkaloids, flavonoids, aldehydes, tannins, phenols, etc. In addition to the antioxidant and antibacterial properties of these phytochemicals, they can form AgNPs by reducing metal salts (Arshad et al., 2024). Other biological sources including bacterial and fungi enzymes or metabolites have also been explored for this purpose (Raj et al., 2022).

The present study reports successful biosynthesis of AgNPs using metabolites from *Pseudomonas* sp. Z9.3. AgNP displayed a clear surface plasmon resonance peak at 400-450 nm, indicative of the reduction of Ag^+^ to Ag^0^ (Hossain et al., 2019; Wan Mat Khalir et al., 2020). Despite the variety of shapes AgNPs can take, bacterial-based reduction typically results in a spherical shape. The size and shape of AgNPs varies greatly depending on the strain of bacteria used for the biosynthesis, as revealed for *Bacillus amyloliquefaciens* (Hossain et al., 2023) or *B. subtilis* (El-Bendary et al., 2021), with average diameters of 20 nm or higher and a variety of shapes. Reports about *Pseudomonas* indicate that AgNPs synthetized by strains in this genus are always spherical and smaller than those produced by *Bacillus*. *P. canadensis* produce AgNPs of slightly higher diameters (21 to 52 nm; Ghasemi et al., 2023) than *P. alloputida* B003, in which size ranged from 7 to 19 nm in diameter (Pernas-Pleite et al., 2022), in line with our average size 13.32 ± 0.4 nm for S1-9 and 8.24 ± 0.26 nm for S4-7. Interestingly, the influence of pH seems relevant in the process as pH9 produces AgNPs of larger diameters, despite the lower concentration of silver (1 volume of 5 mM AgNO_3_) suggesting a richer corona of organic matter coating the NPs. The difference in the size and shape of the synthesized NPs mainly depends on the synthesis conditions (temperature, time, pH and AgNO_3_ concentration), which will further affect the formation of particles.

In addition to their spherical shape, the reduced metal forms a crystal structure corresponding to face-centered cubic AgCl, as indicated by XRD analysis, probably due to the high NaCl content in the culture media (Plokhovska et al., 2025). Furthermore, the organic composition of the shell, the surface coating that leads to effective stabilization of the produced NPs, reveals a unique composition of bacterial metabolites present in supernatants. A comparison between the raw culture media and the bacterial supernatant shows differences in organic matter caused by bacterial metabolism. Furthermore, comparing supernatant to AgNPs reveals a similar but not identical profile, highlighting the selective metabolites capable of reducing the metal and stabilizing the nanoparticles. Raw culture media was unable to reduce silver (data not shown). Characterization of organic matter in the shell of other NP indicates different types of compounds, but in most cases proteins and lipids are found to be present (Hossain et al., 2019; Pernas-Pleite et al., 2022). Despite this general trend, the composition of the AgNPs produced in this study is unique, as it reflects the metabolites from *Pseudomonas* sp. Z9.3, which have unique biological activities, based on the bacterial beneficial effects on plants.

In addition to potential effects on plants, its metal nuclei, silver, has been already employed as an antibacterial agent that inhibits microorganisms of all types, including fungi, bacteria, and viruses (Rai et al., 2021). Our AgNPs were able to restrict growth of pathogenic strains. According to the Standard Antibacterial test “SNV 195920-1992,” molecules showing more than 1 mm microbial zone inhibition can be considered as good antibacterial agents (Singla et al., 2022; Wan Mat Khalir et al., 2020). Our results, suggest that our bio-synthesized AgNPs could be useful as antibacterial agents with biomedical applications, and may have potential to be used in the agricultural field (Khan et al., 2023). Several mechanisms have been reported for the antimicrobial activity of AgNPs, but the exact mechanism has not been unveiled yet. The most widely accepted mechanism, is that positively charged silver ions can interact with negatively charged phosphorus or sulfur contained in biomacromolecules compounds (proteins and nucleic acid), causing structural changes and deformation of bacterial cell wall and membrane that leads into the disruption of several metabolic processes, followed by cell death (More et al., 2023). However, this mechanism assumes that the Ag^0^ in the NP nuclei us liberated and it is transformed in its oxidized form and therefore, becomes toxic upon Ag^+^ release. Our hypothesis does not support these mechanisms, as the Ag^0^ of biosynthesized NPs is stabilized by bacterial organic matter in the corona, and therefore, remains reduced.

**Figure 5:**
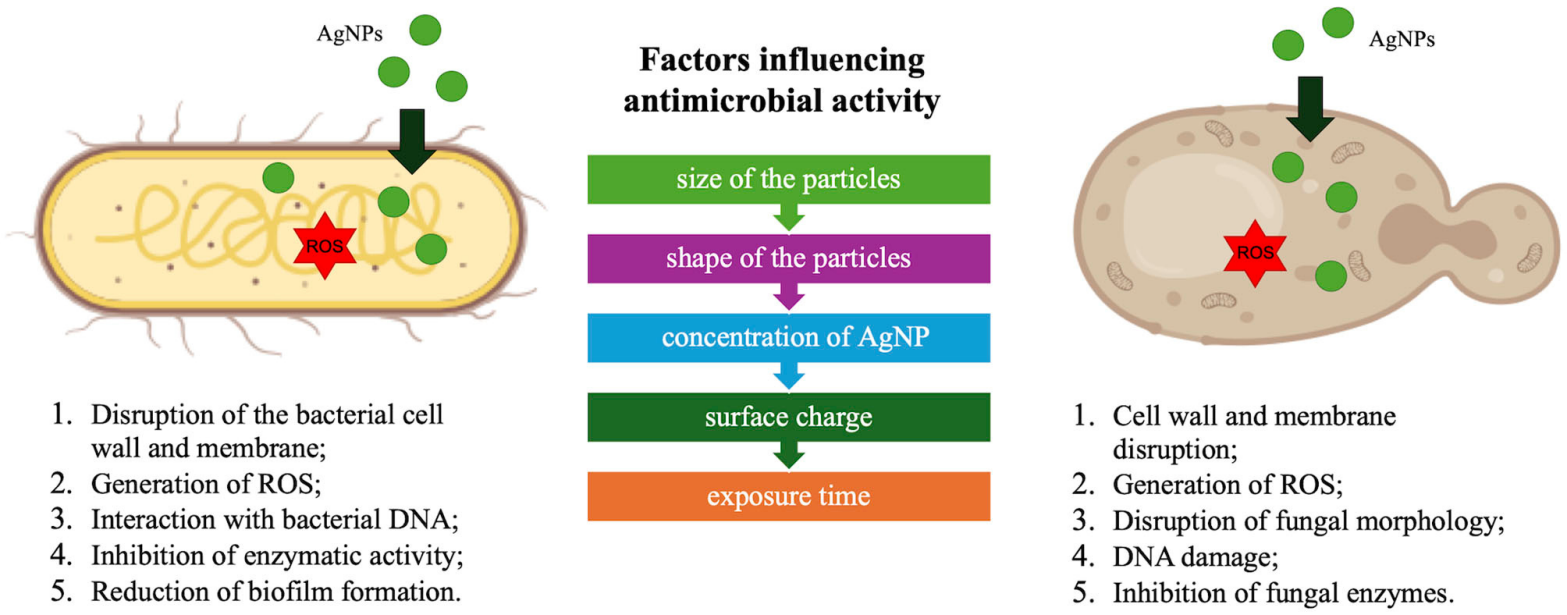
Proposed antimicrobial effects of AgNPs.

The great diversity of AgNPs is due to the biomaterials and conditions used, and represents an additional difficulty to decipher a single mechanism of action. The mechanism of action proposed is that AgNPs change the permeability of the pathogen's membrane and leads to NP accumulation inside the cell (Figure 5). Specifically, AgNPs exhibit antimicrobial effects against both bacteria and fungi by disrupting cell walls and membranes, generating ROS that damage DNA, and inhibiting enzymatic activity. However, the proposed mechanisms of action differ slightly: in bacteria, in addition to the previous mechanisms, NPs may reduce biofilm formation, while in fungi, they alter cell morphology, leading to cell death. It is known that ROS can play an important role in deoxyribonucleic acid modification and cause different issues related to DNA (damaged DNA replication and cell propagation; Rai et al., 2021; Mussin and Giusiano, 2022). As noted in our study, the biosynthesized AgNPs from the beneficial strain *Pseudomonas* Z9.3 have unique properties (size, shape, and coating agents) and may be good candidates for potential application as antimicrobial agents.

Irrespective of the mechanisms involved, the AgNPs biosynthesized in the present study have a strong antibacterial activity against human pathogenic bacteria, phytopathogenic bacteria and phytopathogenic fungi. Inhibitory activity was concentration-dependent, and adjusting the pH of supernatants before nucleation significantly influenced the results. Pathogen growth inhibition was consistently more intense with AgNPs than with the equivalent concentration of AgNO_3_, suggesting that bacterial metabolites play a crucial role in enhancing the bactericidal effect. The bactericidal activity of bacterial-synthesized AgNPs against several plant pathogens has been reported previously. Despite variations in biosynthetic conditions, the effective concentrations reported fall within similar ranges (Ghasemi et al., 2023; Marpu et al., 2017).

The present study reinforces the potential of AgNPs to control phytopathogenic bacteria and phytopathogenic fungi outbreaks. The antifungal effects of Z9.3 AgNPs make them ideal candidates to be used as fungicides against plant pathogens (Abdel-Hafez et al., 2016; Subedi et al., 2021), despite the different sensitivity shown by each fungal species. *Alternaria* and *Stemphylium* sp. were most sensitive (74.97 and 66.3%, respectively) while *Fusarium* and *Rhizopus* sp. were less sensitive (45.62 and 32.68%, respectively); in either case, if this activity is kept in the field, 30% reduction of *Rhizopus* sp. spread is really interesting for producers specially under organic farming. The proposed mechanisms for antifungal activity have been reported at several levels from preventing mycelium growth, to germinating spores, developing germ tubes, or lowering fungal-toxin production, or inhibiting biofilm formation (Hussain et al., 2023; Mansoor et al., 2021).

## 5 Conclusions

In summary, the present study highlights the unique and novel properties of the AgNPs synthesized using the filtrated culture of *Pseudomonas* sp. Z9.3. These AgNP demonstrated superior antibacterial and antifungal efficacy compared to equivalent concentration of Ag. The unique combination of a 13.8 nm spherical shape and a distinctive organic corona composition sets these NPs apart, enhancing their stability and bioactivity. AgNP effectively inhibited the growth of bacterial pathogens and fungal phytopathogens, including *Stemphylium, Alternaria*, and *Fusarium* species. Based on the obtained results of antimicrobial activity, the potential use of these AgNPs as a means of control against human and plant pathogens is closer to practical application specifically in the agronomic environment within sustainable agricultural practices.

## Data Availability

The datasets generated and/or analyzed during the current study are available in the Zenodo repository, https://doi.org/10.5281/zenodo.14097742.
