# Supplementary material for: AgNPs biosynthesized from Pseudomonas Z9.3 metabolites as antimicrobial agents against bacterial and fungal pathogens
Source: Front Microbiol. 2025 Apr 7;16:1565689. doi: 10.3389/fmicb.2025.1565689 (PMC12009911; doi:10.3389/fmicb.2025.1565689)
Supplement: Supplementary file 1 [file Image_1.PDF]

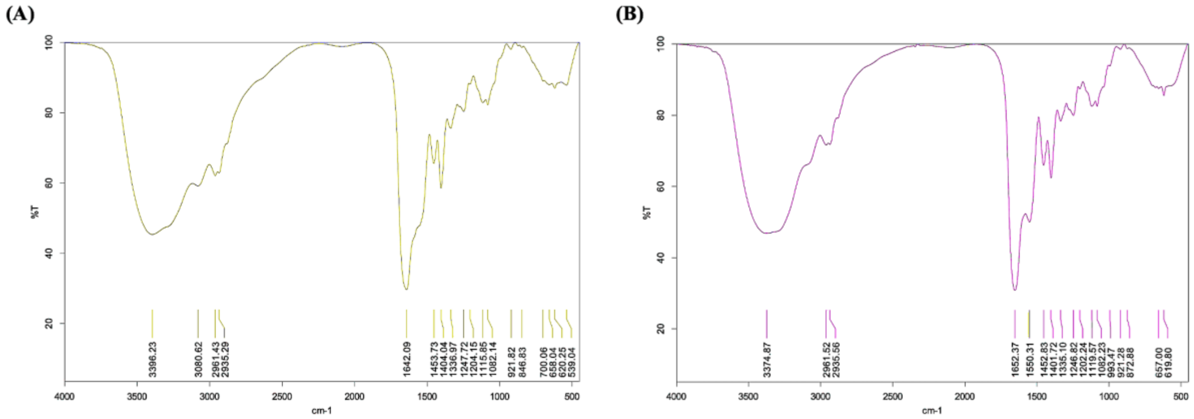

Supplementary Figure S1. FTIR spectra of the components on the surface of nutrient broths **(A)** and the bacterial filtrate **(B)** of *Pseudomonas* Z9.3 before synthesis.
