# Supplementary material for: AgNPs biosynthesized from Pseudomonas Z9.3 metabolites as antimicrobial agents against bacterial and fungal pathogens
Source: Front Microbiol. 2025 Apr 7;16:1565689. doi: 10.3389/fmicb.2025.1565689 (PMC12009911; doi:10.3389/fmicb.2025.1565689)
Supplement: Supplementary file 2 [file Table_1.DOCX]

**Supplementary Table S1**. Antibacterial efficacy of AgNPs against human pathogenic bacteria.

| Bacteria species | **Mean diameter of growth inhibition zone (mm)** | | | | | | | | | | |
| --- | --- | --- | --- | --- | --- | --- | --- | --- | --- | --- | --- |
|  | ***S. aureus*** | ***S. epidermidis*** | | ***Enterococcus*** | | ***Salmonella*** | | ***P. aeruginosa*** | | ***E. coli*** | |
| **S1/pH 9 – amount of AgNPs (%)** | | | | | | | | | | | |
| 10 | 9.74 ± 0.14 | | 10.96 ± 0.17 | | 8.27 ± 0.24 | | 10.54 ± 0.16 | | 11.67 ± 0.12 | | 10.07 ± 0.07 |
| 25 | 11.62 ± 0.20 | | 12.28 ± 0.27 | | 10.67 ± 0.30 | | 11.52 ± 0.32 | | 12.47 ± 0.16 | | 12.14 ± 0.11 |
| 50 | 12.39 ± 0.14 | | 12.67 ± 0.24 | | 11.97 ± 0.27 | | 12.44 ± 0.26 | | 14.32 ± 0.24 | | 12.48 ± 0.07 |
| 75 | 12.58 ± 0.15 | | 12.93 ± 0.11 | | 12.41 ± 0.30 | | 12.69 ± 0.29 | | 14.79 ± 0.12 | | 12.64 ± 0.22 |
| 100 | 14.63 ± 0.27 | | 13.46 ± 0.32 | | 12.85 ± 0.30 | | 13.25 ± 0.30 | | 14.93 ± 0.32 | | 13.28 ± 0.43 |
| AgNO_3_/S1 | 9.28 ± 0.17 | | 6.96 ± 0.22 | | 8.35 ± 0.30 | | 8.51 ± 0.09 | | 9.57 ± 0.30 | | 8.91 ± 0.35 |
| **S4/pH 7 – amount of AgNPs (%)** | | | | | | | | | | | |
| 10 | 7.72 ± 0.22 | | 7.80 ± 0.10 | | 7.49 ± 0.04 | | 7.50 ± 0.16 | | 7.17 ± 0.04 | | 7.72 ± 0.12 |
| 25 | 8.27 ± 0.21 | | 8.50 ± 0.21 | | 7.54 ± 0.13 | | 8.71 ± 0.27 | | 8.25 ± 0.14 | | 8.87 ± 0.26 |
| 50 | 8.57 ± 0.28 | | 9.08 ± 0.11 | | 9.14 ± 0.21 | | 9.09 ± 0.20 | | 8.75 ± 0.22 | | 9.34 ± 0.11 |
| 75 | 10.04 ± 0.12 | | 10.37 ± 0.10 | | 9.27 ± 0.23 | | 9.21 ± 0.29 | | 9.83 ± 0.15 | | 9.90 ± 0.13 |
| 100 | 11.11 ± 0.13 | | 10.53 ± 0.16 | | 10.55 ± 0.19 | | 10.49 ± 0.13 | | 10.73 ± 0.25 | | 11.01 ± 0.33 |
| AgNO_3_/S4 | 9.53 ± 0.01 | | 7.29 ± 0.36 | | 9.32 ± 0.32 | | 9.02 ± 0.09 | | 11.00 ± 0.50 | | 9.35 ± 0.27 |
