# Supplementary material for: AgNPs biosynthesized from Pseudomonas Z9.3 metabolites as antimicrobial agents against bacterial and fungal pathogens
Source: Front Microbiol. 2025 Apr 7;16:1565689. doi: 10.3389/fmicb.2025.1565689 (PMC12009911; doi:10.3389/fmicb.2025.1565689)
Supplement: Supplementary file 3 [file Table_2.DOCX]

**Supplementary Table S2**. Antibacterial efficacy of AgNPs against phytopathogenic bacteria.

|  | **Mean diameter of growth inhibition zone (mm)** | | | | | |
| --- | --- | --- | --- | --- | --- | --- |
|  | **NP S1-9** | | | **NP S4-7** | | |
| Amount of NPs (%) | *X. campestris pv oryzae* | *X. campestris pv tomato* | *P. syringae DC3000* | *X. campestris pv oryzae* | *X. campestris pv tomato* | *P. syringae DC3000* |
| *10* | 10.31 ± 0.27 | 10.0 ± 0.35 | 8.64 ± 0.04 | 9.85 ± 0.37 | 11.29 ± 0.19 | 9.35 ± 0.12 |
| *25* | 10.34 ± 0.14 | 10.79 ± 0.27 | 9.66 ± 0.28 | 10.51 ± 0.18 | 11.67 ± 0.27 | 9.86 ± 0.32 |
| *50* | 11.39 ± 0.3 | 11.39 ± 0.19 | 10.33 ± 0.26 | 10.55 ± 0.18 | 11.99 ± 0.21 | 11.10 ± 0.26 |
| *75* | 11.42 ± 0.11 | 11.57 ± 0.16 | 11.11 ± 0.18 | 10.84 ± 0.4 | 12.31 ± 0.3 | 11.26 ± 0.19 |
| *100* | 11.56 ± 0.14 | 12.10 ± 0.2 | 11.20 ± 0.25 | 11.39 ± 0.27 | 12.78 ± 0.19 | 11.42 ± 0.13 |
| AgNO_3_ | 7.22 ± 0.28 | 7.18 ± 0.19 | 7.09 ± 0.07 | 9.04 ± 0.25 | 7.21 ± 0.08 | 7.31 ± 0.42 |
